# The early and mid-term outcomes of acute type A aortic dissection patients with ECMO

**DOI:** 10.3389/fcvm.2024.1509479

**Published:** 2024-12-20

**Authors:** Qingqing Meng, Hongkai Jiang, Tianbao Li, Shanwen Pang, Chengbin Zhou, Huanlei Huang, Tucheng Sun, Jinlin Wu

**Affiliations:** Department of Cardiac Surgery, Guangdong Cardiovascular Institute, Guangdong Provincial People’s Hospital (Guangdong Academy of Medical Sciences), Southern Medical University, Guangzhou, China

**Keywords:** acute type A aortic dissection, extracorporeal membrane oxygenation (ECMO), postcardiotomy cardiogenic shock (PCS), aorta, outcome

## Abstract

**Background:**

Acute type A aortic dissection (ATAAD) poses significant challenges in cardiovascular management due to its high morbidity and mortality rates. Postcardiotomy cardiogenic shock (PCS) is a severe complication following ATAAD repair that complicates postoperative recovery. Extracorporeal membrane oxygenation (ECMO) has emerged as a potential life-saving intervention in this context, yet the specific outcomes related to ECMO in ATAAD patients remain insufficiently studied.

**Methods:**

This retrospective single-center study reviewed the medical records of 479 patients who underwent ATAAD surgery from September 2017 to June 2021. Patients were stratified into those requiring postoperative ECMO support and those who did not. Data collected included demographics, operative details, and postoperative outcomes.

**Results:**

Of the cohort, 19 patients (4.0%) required ECMO support. The ECMO group exhibited significantly higher mortality rates (57.9% vs. 5.4%, *p* < 0.001) and increased complications, including a higher rate of continuous renal replacement therapy (84.2% vs. 24.3%, *p* < 0.001) and prolonged ICU stays (14.5 days vs. 7.6 days, *p* = 0.009). Survival analysis demonstrated a stark contrast in 3-year survival rates, with 36.8% for the ECMO group vs. 92.8% for non-ECMO patients (*p* < 0.001).

**Conclusions:**

ECMO can be a crucial intervention for ATAAD patients suffering from PCS; however, it is associated with significantly higher mortality and complications. Despite lower long-term survival rates compared to non-ECMO patients, ECMO may offer a survival benefit as a salvage therapy. Interpretation is limited by the retrospective single-center design, small ECMO cohort size, and lack of post-discharge quality-of-life data.

## Background

Acute type A aortic dissection (ATAAD) remains one of the most challenging cardiovascular emergencies, associated with high morbidity and mortality rates despite advancements in surgical techniques and perioperative care (1, 2). The International Registry of Acute Aortic Dissection (IRAD) reports in-hospital mortality rates ranging from 15% to 30%, highlighting the severity of this condition (2). Postcardiotomy cardiogenic shock (PCS) following ATAAD repair further compounds the risk, presenting a formidable challenge in postoperative management.

Extracorporeal membrane oxygenation (ECMO) has emerged as a valuable tool for managing PCS in cardiac surgery patients, providing temporary mechanical circulatory support to maintain end-organ perfusion and allow for myocardial recovery (3, 4). While several studies have investigated ECMO outcomes in general cardiac surgery populations, reporting survival to hospital discharge rates between 20% and 50%, the specific outcomes of ECMO therapy in patients with PCS following ATAAD repair remain largely unexplored (4, 5).

The complex nature of ATAAD surgery, including extended cardiopulmonary bypass times, deep hypothermic circulatory arrest, and the potential for malperfusion syndromes, may pose unique challenges for postoperative ECMO management (5). Moreover, the residual dissected aorta and the risk of ongoing malperfusion could potentially impact the efficacy and complications of ECMO support in this patient population. This study aims to investigate the early and mid-term outcomes of ECMO support in patients with PCS following ATAAD repair.

## Methods

### Study design and patient population

This retrospective, single-center study adhered to the principles of the Declaration of Helsinki (2013 revision) (6) and received approval from the Institutional Review Board of Guangdong Provincial People's Hospital (ID: 2019-842H-1) on July 6th, 2021. The observational nature of the study allowed for a waiver of informed consent.

We conducted a comprehensive review of medical records for all patients who underwent ATAAD surgery at our institution from September 2017 to June 2021. Patients were stratified into two cohorts based on their requirement for post-operative ECMO support.

### Data collection and outcomes

A thorough examination of electronic medical records and hospital charts provided anthropometric, radiologic, laboratory, and operative data. Follow-up information was gathered through a combination of clinical visits and telephone communications.

Primary outcomes encompassed operative mortality (defined as in-hospital or 30-day postoperative death), stroke, paraplegia, and need for continuous renal replacement therapy (CRRT). Secondary outcomes included re-exploration for bleeding, delayed chest closure, acute lung injury (defined by an oxygenation index ≤100), hospital and intensive care unit (ICU) length of stay, and duration of mechanical ventilation. Malperfusion syndrome was categorized using the Penn classification (7).

### Surgical approach and ECMO protocol

Our surgical strategy for ATAAD typically involved total arch replacement with a tetrafurcate graft and frozen elephant trunk implantation. Cardiopulmonary bypass was generally established via right axillary artery cannulation, employing moderate hypothermic circulatory arrest and selective antegrade cerebral perfusion for neuroprotection.

Venoarterial ECMO was initiated intraoperatively for patients exhibiting refractory PCS, characterized by difficulty weaning from cardiopulmonary bypass with an inotropic equivalent score exceeding 50 (8). The ECMO circuit comprised a centrifugal pump and microporous membrane oxygenator. The axillary artery was the preferred site for arterial cannulation, while venous access was often obtained via the femoral vein.

ECMO management aimed to maintain a mean arterial pressure above 65 mmHg and central venous oxygen saturation exceeding 65%. Heparinization commenced 24 h post-operation, targeting an activated clotting time of 160–180 s. In cases of severe active bleeding, heparin administration was reconsidered. Daily sedation interruption at 8 AM allowed for neurological assessment and family visitation. ECMO weaning was contemplated when patients demonstrated cardiopulmonary stability, characterized by an inotropic equivalent score below 10 and the ability to reduce ECMO flow to less than 1.0 L/min.

### Statistical analysis

We employed the Kolmogorov-Smirnov test to assess normality of continuous variables, presenting them as mean ± standard deviation or median with interquartile range (IQR) as appropriate. Categorical variables were expressed as frequencies and percentages. Group comparisons utilized independent *t*-tests or Mann-Whitney *U*-tests for continuous variables, and Chi-square or Fisher's exact tests for categorical variables.

Survival analysis was performed using the Kaplan-Meier method with log-rank test for group comparisons. Logistic regression identified risk factors for operative mortality, presenting results as odds ratios (OR) with 95% confidence intervals (CI). Statistical significance was set at *p* < 0.05 (two-tailed). All analyses were conducted using R software (version 3.5.1).

## Results

Our single-center cohort included 479 ATAAD patients, of whom 19 (4.0%) required postoperative ECMO support. Baseline characteristics are shown in Table 1. The ECMO group had a significantly higher proportion of Penn Ab classification (84.2% vs. 62.0%, *p* = 0.085) and lower proportion of Penn Aa classification (10.5% vs. 35.4%, *p* = 0.046) compared to the non-ECMO group. The ECMO group also had significantly higher white blood cell counts (14.5 vs. 12.4 *10^9/L, *p* = 0.014) and D-dimer levels (20,000.0 vs. 9,855.0 ng/ml, *p* = 0.001).

**Table 1:** Baseline characteristics of ATAAD patients With and without ECMO support.

Variables	Overall	Non-ECMO	ECMO	*p* value
*n*	479	460	19	
Male (%)	404 (84.3)	389 (84.6)	15 (78.9)	0.518
Age [year, median (IQR)]	52.0 [45.0, 60.0]	52.0 [45.0, 60.2]	51.0 [45.5, 58.5]	0.984
Height [m, median (IQR)]	1.7 [1.6, 1.7]	1.7 [1.6, 1.7]	1.7 [1.7, 1.7]	0.401
Weight [kg, median (IQR)]	70.0 [63.0, 79.0]	70.0 [62.5, 79.0]	72.0 [66.5, 75.5]	0.467
BMI (kg/m^2, median [IQR])	24.5 [22.2, 27.1]	24.5 [22.2, 27.1]	24.5 [22.7, 25.4]	0.837
DeBakey II (%)	49 (10.2)	49 (10.7)	0 (0.0)	0.242
Penn Aa (%)	165 (34.4)	163 (35.4)	2 (10.5)	0.046
Penn Ab (%)	301 (62.8)	285 (62.0)	16 (84.2)	0.085
Penn Ab&c (%)	11 (2.3)	10 (2.2)	1 (5.3)	0.921
Penn Ac (%)	2 (0.4)	2 (0.4)	0 (0.0)	1.000
Aortic regurgitation (%)	157 (32.8)	148 (32.2)	9 (47.4)	0.211
Smoking (%)	163 (34.0)	159 (34.6)	4 (21.1)	0.323
Pulmonary artery hypertension (%)	35 (7.3)	34 (7.4)	1 (5.3)	1.000
Hypertension (%)	326 (68.1)	310 (67.4)	16 (84.2)	0.140
Coronary artery disease (%)	46 (9.6)	42 (9.1)	4 (21.1)	0.099
Marfan syndrome (%)	25 (5.2)	25 (5.4)	0 (0.0)	0.614
Hx of cardiovascular surgery (%)	38 (7.9)	38 (8.3)	0 (0.0)	0.386
Diabetes (%)	8 (1.7)	8 (1.7)	0 (0.0)	1.000
COPD (%)	45 (9.4)	42 (9.1)	3 (15.8)	0.408
White blood cell count (×10^9^/L, median [IQR])	12.5 [10.2, 15.2]	12.4 [10.1, 15.2]	14.5 [12.8, 15.9]	0.014
Hemoglobin [g/L, median (IQR)]	133.0 [123.0, 145.5]	133.0 [123.0, 145.2]	136.0 [126.0, 145.5]	0.374
Platelet count (×10^9^/L, median [IQR])	184.0 [151.5, 228.0]	184.0 [152.0, 228.0]	177.0 [137.0, 226.0]	0.369
D-dimer [ng/ml, median (IQR)]	10,120.0 [3,435.0, 20,000.0]	9,855.0 [3,360.0, 20,000.0]	20,000.0 [16,080.0, 20,000.0]	0.001

ATAAD, acute type A aortic dissection; ECMO, extracorporeal membrane oxygenation; BMI, body mass index; IQR, interquartile range; Hx, history; COPD, chronic obstructive pulmonary disease.

Operative data are presented in Table 2. The ECMO group had significantly longer operation times (564.0 vs. 465.0 min, *p* < 0.001), cardiopulmonary bypass times (310.0 vs. 238.5 min, *p* < 0.001), and aortic cross-clamp times (167.0 vs. 129.0 min, *p* < 0.001). They also had a higher rate of concomitant coronary artery bypass grafting (36.8% vs. 5.7%, *p* < 0.001).

**Table 2:** Operative data of ATAAD patients With and without ECMO support.

Variables	Overall	Non-ECMO	ECMO	*p* value
*n*	479	460	19	
Root				
Root_replacement	119 (24.8)	109 (23.7)	10 (52.6)	
Root_sparing	322 (67.2)	315 (68.5)	7 (36.8)	
Wheat	38 (7.9)	36 (7.8)	2 (10.5)	
Arch (%)				1.000
Arch_Sparing	5 (1.0)	5 (1.1)	0 (0.0)	
Hemiarch	11 (2.3)	11 (2.4)	0 (0.0)	
Hybrid_TAR	2 (0.4)	2 (0.4)	0 (0.0)	
TAR	461 (96.2)	442 (96.1)	19 (100.0)	
CABG (%)	33 (6.9)	26 (5.7)	7 (36.8)	<0.001
FET (%)	463 (96.7)	444 (96.5)	19 (100.0)	1.000
Operation time [min, median (IQR)]	470.0 [420.0, 540.0]	465.0 [415.0, 533.2]	564.0 [500.5, 690.0]	<0.001
CPB time [min, median (IQR)]	241.0 [210.5, 280.0]	238.5 [209.8, 275.0]	310.0 [280.0, 371.0]	<0.001
ACC time [min, median (IQR)]	132.0 [103.0, 160.0]	129.0 [103.0, 159.0]	167.0 [150.0, 195.0]	<0.001
MHCA time [min, median (IQR)]	20.0 [16.0, 24.0]	20.0 [16.0, 24.0]	20.0 [15.0, 27.0]	0.463
MHCA (%)	314 (65.6)	302 (65.7)	12 (63.2)	0.809

ATAAD, acute type A aortic dissection; ECMO, extracorporeal membrane oxygenation; TAR, total arch replacement; CABG, coronary artery bypass graft; FET, frozen elephant trunk; CPB, cardiopulmonary bypass; ACC, aortic cross-clamp; MHCA, moderate hyperthermic cardiac arrest; IQR, interquartile range.

Surgical outcomes are shown in Table 3. The ECMO group had significantly higher rates of mortality (57.9% vs. 5.4%, *p* < 0.001), continuous renal replacement therapy (84.2% vs. 24.3%, *p* < 0.001), and delayed chest closure (63.2% vs. 5.4%, *p* < 0.001). They also had longer ICU stays (14.5 vs. 7.6 days, *p* = 0.009) and mechanical ventilation times (298.0 vs. 96.0 h, *p* < 0.001). Among the 19 patients who received ECMO support, major ECMO-related complications included bleeding requiring re-exploration (*n* = 1, 5.3%), limb ischemia (*n* = 2, 10.5%), and access site infections (*n* = 2, 10.5%).

**Table 3:** Operative outcomes of ATAAD patients with and without ECMO support.

Variables	Overall	Non-ECMO	ECMO	*p* value
*n*	479	460	19	
Mortality (%)	36 (7.5)	25 (5.4)	11 (57.9)	<0.001
Stroke (%)	27 (5.6)	24 (5.2)	3 (15.8)	0.084
Paraplegia (%)	21 (4.4)	21 (4.6)	0 (0.0)	1.000
CRRT (%)	128 (26.7)	112 (24.3)	16 (84.2)	<0.001
Re-exploration (%)	7 (1.5)	6 (1.3)	1 (5.3)	0.248
Delayed chest closure (%)	37 (7.7)	25 (5.4)	12 (63.2)	<0.001
Acute lung injury (%)	132 (27.6)	123 (26.7)	9 (47.4)	0.065
Hospital stay [day, median (IQR)]	23.0 [18.0, 31.0]	23.0 [18.0, 31.0]	25.0 [9.5, 42.5]	0.733
ICU stay [day, median (IQR)]	7.7 [4.8, 12.3]	7.6 [4.8, 11.9]	14.5 [6.0, 29.3]	0.009
Mechanical ventilation time [hour, median (IQR)]	97.0 [47.0, 169.0]	96.0 [46.0, 162.2]	298.0 [143.0, 469.5]	<0.001

ATAAD, acute type A aortic dissection; ECMO, extracorporeal membrane oxygenation; CRRT, continuous renal replacement therapy; ICU, intensive care unit; IQR, interquartile range.

**Figure 1:**
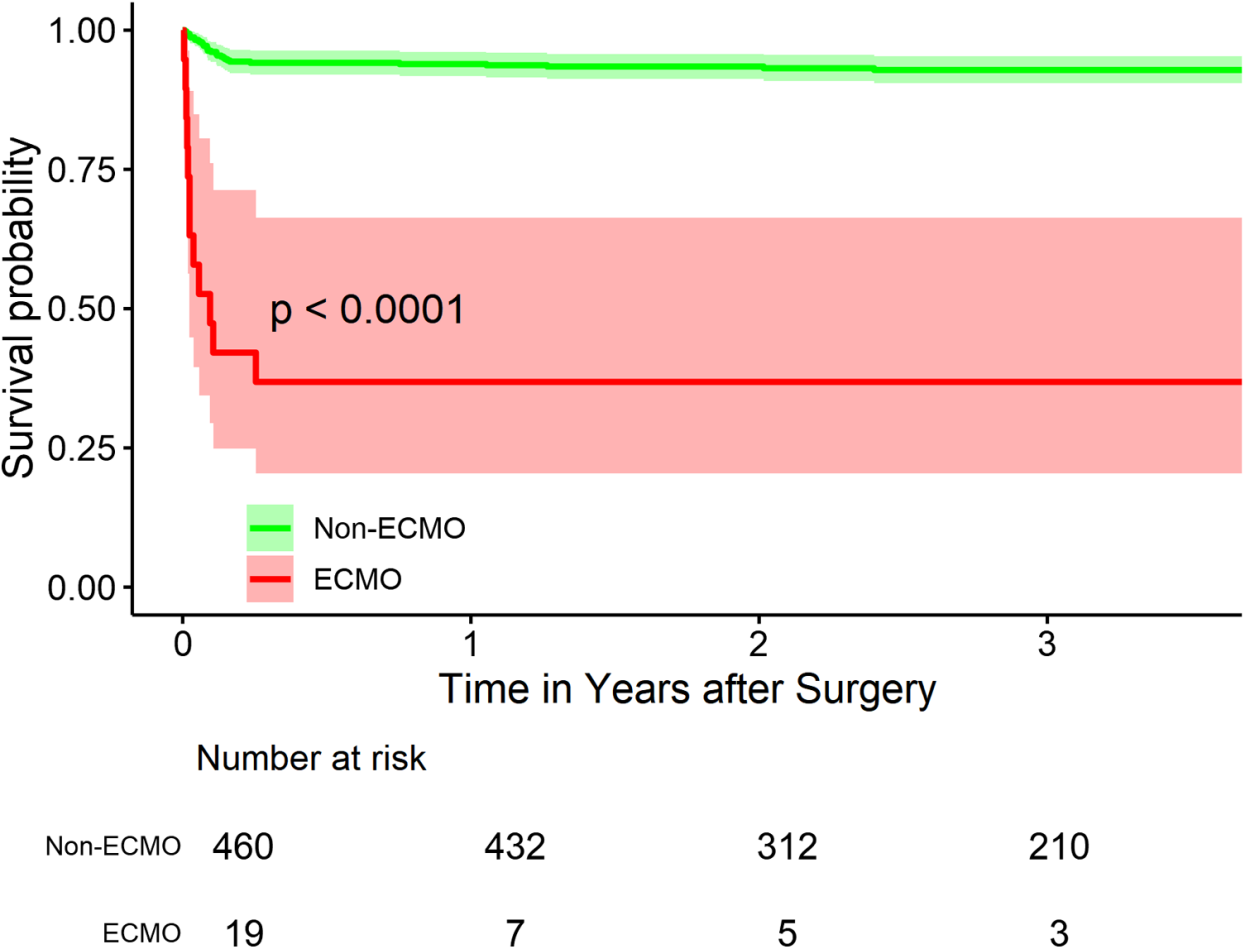
Kaplan-Meier survival curves for ATAAD patients With and without ECMO support. Figure 1 shows the Kaplan-Meier survival curves for acute type A aortic dissection (ATAAD) patients with and without extracorporeal membrane oxygenation (ECMO) support. The survival rates at 1-, 2- and 3-year follow-up were 93.9%, 93.4% and 92.8% respectively for the non-ECMO group, compared to 36.8%, 36.8% and 36.8% for the ECMO group (*p* < 0.001), indicating significantly poorer mid-term survival outcomes in patients requiring ECMO support.

Long-term survival analysis revealed significant differences between the ECMO and non-ECMO groups (Figure 1). The survival rates at 1-, 2- and 3-year follow-up were 93.9%, 93.4% and 92.8% respectively for the non-ECMO group, compared to 36.8%, 36.8% and 36.8% for the ECMO group (*p* < 0.001).

Univariate analysis identified several preoperative and intraoperative predictors of mortality. These included age (OR: 1.05, 95% CI: 1.01–1.09, *p* = 0.009), Penn Aa classification (OR: 0.29, 95% CI: 0.10–0.69, *p* = 0.011), hypertension (OR: 2.48, 95% CI: 1.08–6.73, *p* = 0.047), D-dimer levels (OR: 1.00, 95% CI: 1.00–1.00, *p* = 0.001), concomitant coronary artery bypass grafting (OR: 14.20, 95% CI: 6.26–32.13, *p* < 0.001), operation time (OR: 1.01, 95% CI: 1.00–1.01, *p* < 0.001), cardiopulmonary bypass time (OR: 1.01, 95% CI: 1.01–1.02, *p* < 0.001), and aortic cross-clamp time (OR: 1.02, 95% CI: 1.01–1.03, *p* < 0.001). In the multivariate analysis, after controlling for preoperative and postoperative risk factors, ECMO remained a strong predictor of mortality (OR: 10.5, 95% CI: 2.97- 37.2, *p* < 0.001).

## Discussion

This study provides valuable insights into the outcomes of ECMO support in patients with ATAAD who develop PCS. Our findings reveal that while ECMO can be a life-saving intervention, it is associated with significantly higher mortality rates and complications compared to non-ECMO patients.

The utilization rate of ECMO in our cohort (4.0%) is consistent with previous reports in cardiac surgery populations, which range from 0.6% to 2.9% (3). However, our study focused specifically on ATAAD patients, a high-risk subgroup that may require ECMO more frequently due to the complex nature of the surgery and potential preoperative malperfusion syndromes. Lin et al. reported a higher ECMO utilization rate of 12.3% in their ATAAD cohort, which may reflect differences in patient characteristics or institutional protocols (5).

Our results demonstrate significantly longer operative times, cardiopulmonary bypass durations, and aortic cross-clamp times in the ECMO group. These findings are in line with previous studies that have identified prolonged cardiopulmonary bypass time as a risk factor for PCS and subsequent need for mechanical circulatory support (9, 10). The extended operative times may reflect the technical challenges encountered during surgery, potentially due to more complex aortic pathology or concomitant procedures such as coronary artery bypass grafting, which was more prevalent in our ECMO group. Coronary artery bypass grafting also indicates severe root involvement and coronary malperfusion.

The high mortality rate observed in our ECMO cohort (57.9%) is concerning but not unexpected given the severity of illness in these patients. This rate is comparable to other studies reporting on ECMO use in PCS, with mortality rates ranging from 50% to 80% (11, 12). However, it's important to note that without ECMO support, mortality in this critically ill subgroup would likely approach 100%. The survival benefit of ECMO in this context, while modest, may justify its use as a salvage therapy in carefully selected patients. Interestingly, the plateauing of survival curves after hospital discharge suggests that patients who survive the initial hospitalization may achieve relatively stable long-term outcomes.

Long-term survival analysis revealed a significant difference between ECMO and non-ECMO groups, with 3-year survival rates of 36.8% and 92.8%, respectively. This finding underscores the impact of PCS on not only short-term but also mid-term outcomes. However, it's noteworthy that for ECMO survivors, the survival curve plateaus after the initial hospitalization, suggesting that patients who overcome the acute phase may have a relatively stable long-term prognosis. This observation is consistent with the findings of Lorusso et al., who reported that long-term survival and quality of life in ECMO survivors were comparable to age- and sex-matched general populations (12).

The higher rates of complications in the ECMO group, including renal failure requiring continuous renal replacement therapy and prolonged mechanical ventilation, highlight the complex nature of managing these patients. These complications may be attributed to the severity of the initial insult, the systemic inflammatory response associated with ECMO, and the challenges of anticoagulation in the setting of recent aortic surgery (13). Strategies to mitigate these complications, such as early initiation of renal replacement therapy and lung-protective ventilation strategies, should be considered in the management protocol for these patients.

ECMO offers several unique advantages in the management of post-ATAAD cardiogenic shock compared to other mechanical circulatory support options. Unlike temporary right ventricular assist devices or isolated left ventricular support, ECMO provides comprehensive cardiopulmonary support, particularly beneficial given the frequent occurrence of biventricular failure and respiratory complications in these patients. However, the risks of bleeding and thrombosis are particularly pertinent in post-dissection patients where the need for anticoagulation must be balanced against recent extensive aortic surgery. Looking ahead, the role of ECMO in ATAAD management may evolve with technological advances such as more biocompatible circuits reducing anticoagulation requirements. Additionally, the development of standardized protocols for early ECMO deployment based on objective criteria, rather than as a rescue therapy, may improve outcomes. Future research should focus on identifying predictive factors for ECMO success in this population, potentially through machine learning approaches analyzing preoperative and intraoperative variables.

## Limitations

Our study has several limitations that should be acknowledged. First, the retrospective, single-center design limits the generalizability of our findings and may introduce selection bias. Second, the small sample size of the ECMO group (*n* = 19) reduces the statistical power of our analyses and increases the risk of type II errors. Third, we were unable to account for all potential confounding factors that may influence the decision to initiate ECMO and affect outcomes. Fourth, we do not have detailed information on the quality of life of survivors, which is an important consideration in evaluating the overall benefit of ECMO support.

## Conclusion

In conclusion, our study demonstrates that while ECMO can be a life-saving intervention for ATAAD patients with PCS, it is associated with high mortality and morbidity rates. The long-term survival of ECMO survivors, although lower than non-ECMO patients, suggests that ECMO can be a valuable salvage therapy in carefully selected patients. Future research should focus on improving patient selection criteria, optimizing ECMO management protocols, and developing strategies to mitigate ECMO-associated complications in this high-risk population.

## Data Availability

The raw data supporting the conclusions of this article will be made available by the authors, without undue reservation.
